# Formation of α-tocopherol hydroperoxide and α-tocopheroxyl radical: relevance for photooxidative stress in Arabidopsis

**DOI:** 10.1038/s41598-020-75634-0

**Published:** 2020-11-12

**Authors:** Aditya Kumar, Ankush Prasad, Pavel Pospíšil

**Affiliations:** Department of Biophysics, Centre of the Region Haná for Biotechnological and Agricultural Research, Faculty of Science, Palacký University, Šlechtitelů 27, 783 71 Olomouc, Czech Republic

**Keywords:** Biochemistry, Biological techniques, Biophysics

## Abstract

Tocopherols, lipid-soluble antioxidants play a crucial role in the antioxidant defense system in higher plants. The antioxidant function of α-tocopherol has been widely studied; however, experimental data on the formation of its oxidation products is missing. In this study, we attempt to provide spectroscopic evidence on the detection of oxidation products of α-tocopherol formed by its interaction with singlet oxygen and lipid peroxyl radical. Singlet oxygen was formed using photosensitizer rose bengal and thylakoid membranes isolated from *Arabidopsis thaliana*. Singlet oxygen reacts with polyunsaturated fatty acid forming lipid hydroperoxide which is oxidized by ferric iron to lipid peroxyl radical. The addition of singlet oxygen to double bond carbon on the chromanol head of α-tocopherol forms α-tocopherol hydroperoxide detected using fluorescent probe swallow-tailed perylene derivative. The decomposition of α-tocopherol hydroperoxide forms α-tocopherol quinone. The hydrogen abstraction from α-tocopherol by lipid peroxyl radical forms α-tocopheroxyl radical detected by electron paramagnetic resonance. Quantification of lipid and protein hydroperoxide from the wild type and tocopherol deficient (*vte1*) mutant Arabidopsis leaves using a colorimetric ferrous oxidation-xylenol orange assay reveals that α-tocopherol prevents formation of both lipid and protein hydroperoxides at high light. Identification of oxidation products of α-tocopherol might contribute to a better understanding of the protective role of α-tocopherol in the prevention of oxidative damage in higher plants at high light.

## Introduction

Higher plants convert solar energy into chemical energy in light-driven photosynthetic reactions. The light reactions involve the capture of solar energy by chlorophylls and the splitting of water into protons, electrons and molecular oxygen. The simultaneous occurrence of molecular oxygen with excited chlorophylls is potentially harmful to plants as singlet oxygen (^1^O_2_) might be formed by excitation energy transfer from triplet chlorophyll to molecular oxygen^1–5^. Singlet oxygen addition to the double bond forms lipid hydroperoxide (LOOH) by the ene reaction, whereas the cycloaddition of ^1^O_2_ across the double bond forms endoperoxides. In the presence of oxidized transition metals (e.g. Fe^3+^), LOOH is oxidized to lipid peroxyl radical (LOO^•^). As the latter possess unpaired electron on oxygen atom, it has capability to abstract a hydrogen atom from other adjacent polyunsaturated fatty acid and thus initiates propagation of lipid peroxidation. To prevent lipid peroxidation, higher plants evolved numerous protection mechanisms comprising non-enzymatic and enzymatic antioxidant defense system. In non-enzymatic reactions, a variety of lipophilic antioxidants can either prevent the initiation or cause the termination of lipid peroxidation. Among lipophilic antioxidants, tocochromanols (tocopherols, tocotrienols and plastochromanol-8) and isoprenoid quinones (plastoquinone-9 and plastoquinol-9) play a crucial role in the prevention of lipid peroxidation^6–8^. It was shown that α-tocopherol (α-TOH) prevents lipid peroxidation in both non-radical and radical reactions. In non-radical reactions, α-TOH maintains physical and chemical quenching of ^1^O_2_^9,10^. In the physical quenching, electron transfer from α-TOH to ^1^O_2_ forms singlet exciplex, which undergoes to triplet exciplex by intersystem crossing^11,12^. In the chemical quenching, the oxidation of α-TOH by ^1^O_2_ leads to the formation of α-tocopherol hydroperoxide (α-TOOH) which decomposes to more stable α-tocopherol-quinone (α-TQ)^13,14^. Whereas the formation of α-TQ was shown in the model systems^15,16^, isolated chloroplasts^17^ and leaves^14,18^, no evidence on α-TOOH formation has been provided. In the radical reaction, α-TOH scavenges LOO^• ^. The hydrogen abstraction from α-TOH by LOO^•^ leads to the formation of α-tocopheroxyl radical (α-TO^•^) which might be re-reduced by ascorbate to α-TOH while monodehydroascorbate radical (Asc^•−^) is formed^19–21^. The α-TO^•^ was detected in the model systems^22,23^; however, no evidence on the formation of α-TO^•^ has been provided in thylakoid membranes.

In this study, we provided for the first-time experimental evidence on the formation of α-TOOH and α-TO^•^ in the thylakoid membranes isolated from Arabidopsis plant. We showed that (1) oxidation of α-TOH by ^1^O_2_ forms α-TOOH monitored by fluorescence spectroscopy and (2) oxidation of α-TOH by LOO^•^ is associated with the appearance of α-TO^•^ detected by electron paramagnetic resonance.

## Materials and methods

### Plant material, growth conditions and stress treatments

In the present work, *Arabidopsis thaliana*, WT (Columbia-0) and tocopherol cyclase deficient mutant, *vte1* (lacking tocopherols and plastochromanol-8) were used^24^. Plants were grown using commercially available substrate (Potgrond H, Klasmann- Deilmann Substrate, Germany) in a growing chamber (Photon Systems Instruments, Drásov, Czech Republic) under precise conditions: photoperiod of 8 h light/16 h dark (120 µmol photons m^−2^ s^−1^), temperature of 22 °C and humidity 60%. High light stress was accomplished in AlgaeTron AG 230 (Photon Systems Instruments, Drásov 470, Czech Republic) by illuminating 5–6 weeks old plants to white light (1500 μmol photons m^−2^ s^−1^) for 13 h at a temperature of 8 °C.

### Thylakoid membrane preparation

Thylakoid membranes were isolated from high light exposed plants using  the protocol developed by Casazza et al.^25^. Harvesting of rosette leaves (0.3–0.5 g) from plants was done followed by floating them on ice-cold water for 10–20 min in the dark and then blotted. Leaves were rapidly homogenized in 10–20 ml of grinding buffer comprising EGTA (5 mM), EDTA (5 mM), MgCl_2_ (5 mM), NaHCO_3_ (10 mM), sorbitol (0.4 M) and tricine/NaOH (20 mM, pH 8.4) and 0.5% (w/v) fatty acid-free BSA was added just before the grinding. Homogenized suspension was filtered through 2 layers of cheesecloth by applying a gentle hand pressure to increase the final thylakoid yield. The filtrate was centrifuged at 2600 g for 3 min at 4 °C, followed by re-suspending the pellet in 10–20 ml of resuspension buffer containing EDTA (2.5 mM), HEPES (20 mM, pH 7.6) MgCl_2_ (5 mM), NaHCO_3_ (10 mM), sorbitol (0.3 M) and 0.5% (w/v) fatty acid-free BSA. Centrifugation was done at 2600 g for 3 min at 4 °C and the pellet was washed again in re-suspension buffer without adding fatty acid-free BSA and then resuspended in 10–20 ml of hypotonic buffer containing EDTA (2.5 mM), MgCl_2_ (5 mM), NaHCO_3_ (10 mM), HEPES (20 mM, pH 7.6) and 0.5% (w/v) fatty acid-free BSA. Thylakoid membranes were collected by centrifugation at 2600 g for 3 min at 4 °C. Finally, the pellet was suspended in a small volume (0.5–1 ml) of resuspension buffer and was stored at -80 °C in the dark until use. The chlorophyll concentrations from thylakoid preparations were calculated from the absorbance at 645 and 663 nm of 80% (v/v) acetone extract, according to Arnon^26^.

### Determination of α-tocopherol and α-tocopherol quinone by HPLC

The amount of α-TOH and α-TQ was assessed by the reverse-phase HPLC analysis using postcolumn reduction with platinum following the protocol described in Nowicka and Kruk^27^. To avoid the auto-oxidation of α-TOH and α-TQ standard, we stored standard at -80 °C in several concentrations. To avoid the auto-oxidation of α-TOH and α-TQ extracted from leaves, we performed the liquid extraction of α-TOH and α-TQ in chilled methanol. We considered the pre-analytical methods mentioned in Giusepponi et al.^28^. α-TOH (30 µM) in the presence of rose bengal (5 µM ) and from thylakoid membranes (750 µg Chl ml^−1^) was extracted in methanol by vortexing (5 min) and centrifuged at 2000 g for 60 s at 4 °C, the supernatant was transferred to HPLC vial using a syringe with needle. Minimum of three independent biological replicates were measured to enable an assessment of significance. Isocratic analysis (0.8 ml min^−1^ at 25 °C) was done using methanol as mobile phase and a LiChrospher 100 RP-18 column (5 µm) LiChroCART 250-4 (Merck, Darmstadt, Germany). Alliance e 2695 HPLC System (Waters Corporation, Milford MA, USA) equipped with a 2998 Photodiode Array (PDA) and a 2475 Fluorescence (FLR) detectors were used. Operation and data processing was performed by Empower 3 Chromatography Data Software (Waters Corporation, Milford MA, USA) (https://www.waters.com/waters/en_US/Empower-3-Chromatography-Data-Software). For determination of α-TOH, fluorescence detection was used (λ_ex_ = 290 nm, λ_em_ = 330 nm). To quantify α-TOH and α-TQ, the calibration curve established in our lab by plotting the peak area at the wavelength for various concentrations of standards was used. α-TOH and α-TQ standards were obtained from Sigma Aldrich GmbH (Germany).

### Determination of α-tocopherol hydroperoxide by fluorescence spectroscopy

The formation of α-TOOH and organic hydroperoxides (ROOH) was measured with a fluorescence spectrophotometer (FP-8000 Series Fluorometer, Easton, MD 21601, USA) using a fluorescent probe swallow-tailed perylene derivative (SPY-LHP) (Dojindo Molecular Technologies Inc. Rockville, MD, USA) ^29,30^. Rose bengal (5 µM) with α-TOH (20 μM) (for α-TOOH detection) and thylakoid membranes (20 µg ml^−1^) (for ROOH detection) were illuminated in the presence of SPY-LHP (2.5 µM) with green and red light (1000 μmol photons m^−2^ s^−1^) for 5 and 15 min, respectively. Green and red light was obtained by a halogen lamp with a light guide (Schott KL 1500, Schott AG, Mainz, Germany) using band-pass interference filter (560 FS10-25) and a long-pass edge interference filter (λ > 600 nm) (Andover Corporation, Salem, NH, USA), respectively. Illumination by green and red light was used to excite rose bengal and chlorophylls and simultaneously avoid SPY-LHP photosensitization. After red light exposure, hydroperoxides were extracted from thylakoid membranes into absolute ethanol and SPY-LHPox fluorescence was measured. SPY-LHPox fluorescence was measured in three biologically distinct samples. The fluorescence emission spectrum was measured at a spectral range between 500 and 650 nm (excitation wavelength, 488 nm). The spectral slit-width for excitation and emission monochromators was 5 nm. The fluorescence intensity at 538 nm was used for the quantification of α-TOOH and ROOH formation.

### Singlet oxygen and radical detection by EPR spectroscopy

The formation of ^1^O_2_, LOO^•^ , α-TO^•^ and Asc^•−^ in the  rose bengal system and ^1^O_2_, organic peroxyl radical (ROO^•^), α-TO^•^ and Asc^•−^ in the thylakoid membranes was measured with EPR spectrometer MiniScope MS400 (Magnettech GmbH, Berlin, Germany). Formation of ^1^O_2_ was detected using hydrophilic probe TMPD (2,2,6,6-tetramethyl-4-piperidone) purchased from Sigma Aldrich GmbH (Germany). The ^1^O_2_ mediated oxidation of diamagnetic TMPD produces paramagnetic 2,2,6,6-tetramethyl-4-piperidone-1-oxyl (TEMPONE) which gives EPR signal detected by EPR spectroscopy. Illumination of rose bengal (5 μM) and thylakoid membranes (250 μg Chl ml^−1^) was done in the presence of 25 mM TMPD and 40 mM phosphate buffer (pH 7.0) at room temperature. Formation of LOO^•^ and ROO^•^ was detected indirectly by DMPO-OL and DMPO-OR adduct EPR signals, respectively using a hydrophilic spin trap DMPO (5,5-dimethyl-pyrroline-1-oxide) purchased from (Sigma Aldrich GmbH, Germany). Illumination of rose bengal (50 μM) with linolenic acid (250 mM) and thylakoid membranes (250 μg Chl ml^−1^) was done in the presence of 50 mM DMPO, 10 mM Fe^3+^ and 40 mM MES-NaOH (pH 6.5). Formation of α-TO^•^ and Asc^•−^ was measured in presence of exogenous α-TOH (50 mM) and sodium ascorbate (50 mM). Illumination of thylakoid membranes was done with white light (1000 µmol m^−2^ s^−1^) using a halogen lamp with a light guide (Schott KL 1500, Schott AG, Mainz, Germany) for the fixed periods and the spectra were recorded. A minimum of three independent biological replicates were measured to assess the significance of measurements. EPR settings were as follows: microwave power, 10 mW; modulation amplitude, 1 G; modulation frequency, 100 kHz; sweep width, 100 G; scan rate, 1.62 G s^−1^.

### Quantification of lipid and protein hydroperoxides by  absorption spectroscopy

To determine LOOH and protein hydroperoxides (POOH) concentration in high light illuminated leaves, lipid and protein extraction was done by following the method of Grintzalis^31^. Leaves harvested from high light illuminated plants were weighed, approximately equal fresh weight of leaves was homogenized in 2–5 ml of 10 mM inorganic phosphate buffer containing 0.5 mM butylated hydroxytoluene (BHT), homogenate was centrifuged at 20,000 g for 10 min at 4 °C and then the filtrate was vigorously vortexed with 2–5 ml of chloroform (CHCl_3_): methanol (CH_3_OH) (2:1) for the lipid extraction. After vigorous vortex, 100% trichloroacetic acid (TCA) (volume of 100% TCA should be 10% of phosphate buffer used for homogenization) was added. The mixture was vortexed for 30–60 s and incubated in ice-cold water for 20 min followed by centrifugation at 20,000*g* for 10 min at 4 °C. After centrifugation, three separate layers (top aqueous layer, middle protein disc and the lower layer containing lipids) were visible. Lower lipid layer was collected in a fresh Eppendorf tube, dried under the nitrogen stream and used for FOX assay. Protein discs were washed with 10% TCA using ultrasonic homogenizer, homogenized protein discs were centrifuged at 20,000*g* for 10 min at 4 °C and the pellet was dissolved in urea (8 M) and used for FOX assay. FOX assay was performed in three biological replicates to confirm the significance of measurements. FOX reagent was prepared fresh by dissolving 15.2 mg xylenol orange (XO) (final concentration 2 mM) in 5 ml of 0.5 M H_2_SO_4_ and bringing to final 10 ml with distilled H_2_O, 5 mM H_2_SO_4_ was prepared by diluting the stock H_2_SO_4_ with distilled H_2_O, 8 mM Fe^2+^ reagent was prepared by dissolving 31.2 mg ferrous ammonium sulfate in 10 ml of 5 mM H_2_SO_4_. All reagents were prepared fresh before use. Lower lipid layer collected in a fresh Eppendorf tube was dried under the nitrogen stream. The dried lipid was dissolved in absolute methanol and protein pellet dissolved in 8 M urea was mixed with Fe^2+^ and FOX reagent and incubated for 30 min at 30 °C. LOOH and POOH formation was monitored by following the changes in absorbance at 560 nm using UV–Visible Spectrophotometer (UV-510 Thermo Spectronic Unicam, UK).

## Results

### Consumption of α-tocopherol detected by HPLC

**Figure 1:**
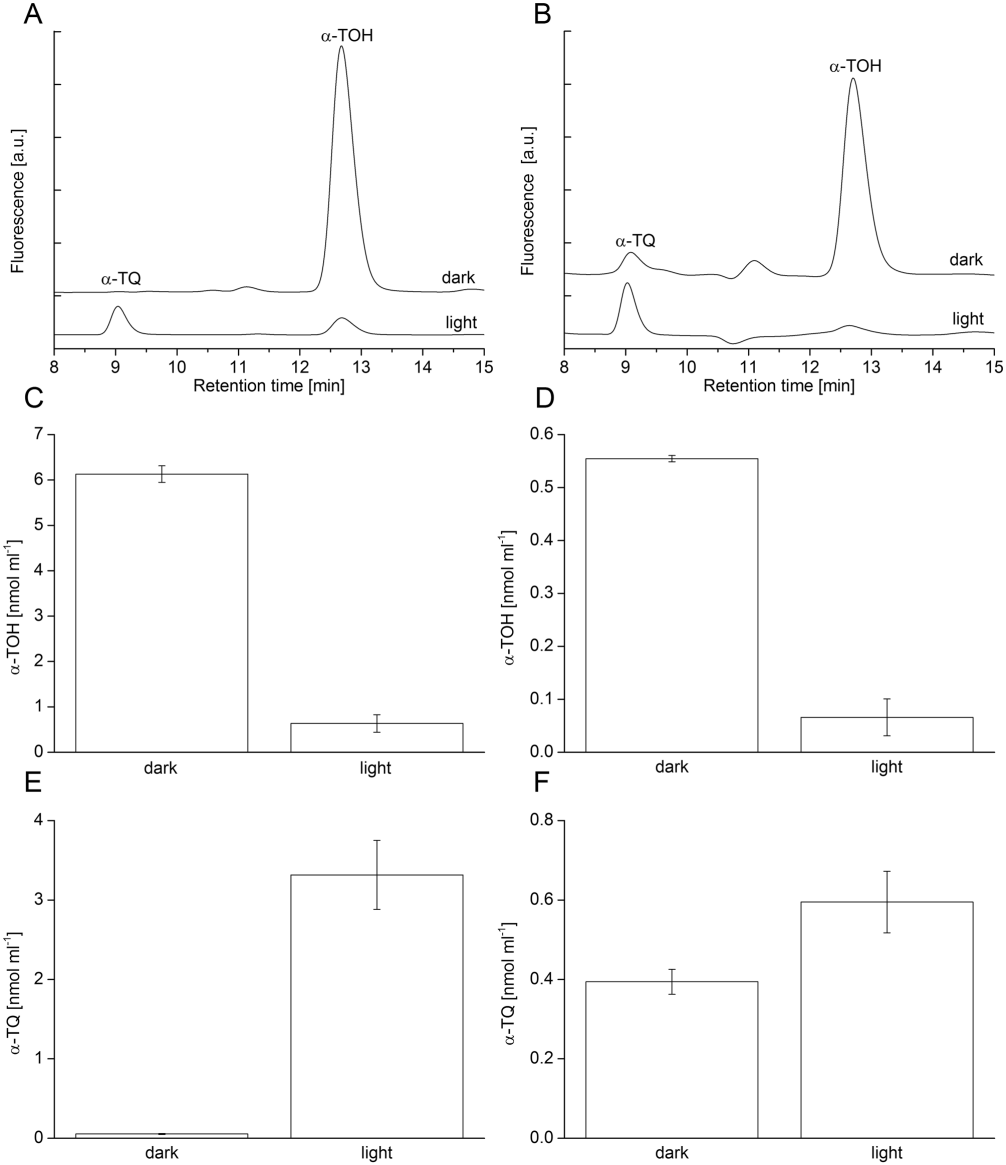
α-Tocopherol and α-tocopherol quinone detection in rose bengal (**A, C, E**) and thylakoid membranes isolated from WT Arabidopsis (**B, D, F**) by high-performance liquid chromatography (HPLC). In A, chromatograms of α-TOH (30 µM) in methanol with rose bengal (5 µM) measured in dark and after white light illumination. In B, chromatograms of α-TOH extracted in methanol from dark and white light illuminated WT thylakoid membranes (750 µg Chl ml^−1^). Rose bengal and thylakoid membranes were illuminated with white light (1000 µmol photons m^−2^ s^−1^) for 5 min and 15 min, respectively. In C-D, quantification of the α-TOH consumption in rose bengal and thylakoid membranes determined as area under a peak at retention time 12.7 min.  In E-F, quantification of α-TQ in rose bengal and thylakoid membranes determined as area under a peak at retention time 9.1 min. Each data point represents the mean ± SD of biological replicates (n = 3).

To monitor the consumption of α-TOH by its oxidation during ^1^O_2_ quenching and LOO^•^ scavenging, the amount of α-TOH was determined by the reverse-phase HPLC analysis using a fluorescence detector. Figure 1 shows the chromatograms of α-TOH added to rose bengal (Fig. 1A) and α-TOH extracted from thylakoid membranes (Fig. 1B). Under the chromatographic conditions used in this study, the observed chromatograms show the peak corresponding to α-TOH at retention time 12.7 min in both rose bengal (Fig. 1A, dark trace) and thylakoid membranes (Fig. 1B, dark trace). When rose bengal and thylakoid membranes were illuminated, suppression of the peak at retention time 12.7 min was observed (Fig. 1A and B, light trace). The concentration of α-TOH in rose bengal (Fig. 1C) and thylakoid membranes (Fig. 1D) in dark was 6.12 ± 0.18 nmol ml^−1^ and 0.55 ± 0.00 nmol ml^−1^, where it decreases to 0.63 ± 0.19 nmol ml^−1^ and 0.06 ± 0.03 nmol ml^−1^ in light, respectively. Rose bengal-photosensitized ^1^O_2_ formation caused complete consumption of α-TOH due to ^1^O_2_ quenching, whereas α-TOH consumption in thylakoid membranes was due to ^1^O_2_ quenching and LOO^•^ scavenging.

### Singlet oxygen quenching by α-tocopherol monitored by EPR spectroscopy

**Figure 2:**
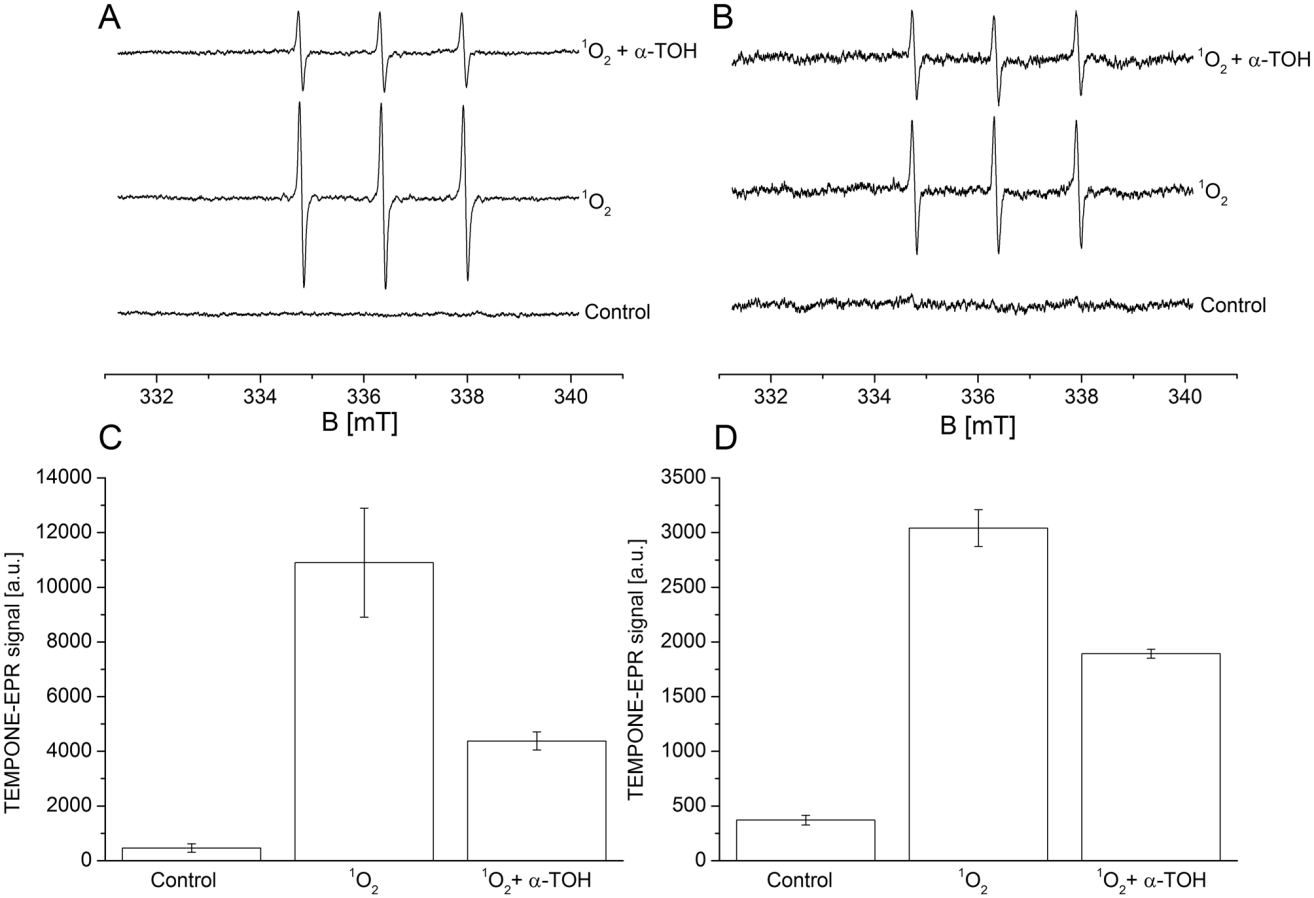
Singlet oxygen formation in rose bengal (**A, C**) and thylakoid membranes isolated from WT Arabidopsis (**B, D**) monitored by electron paramagnetic resonance (EPR) spectroscopy using the spin probe TMPD. In A-B, TEMPONE EPR spectra were recorded in the control  and illuminated rose bengal (5 µM) or thylakoid membranes (250 µg Chl ml^−1^) in the presence of TMPD (25 mM) and phosphate buffer (40 mM, pH 7.0). Illumination of rose bengal and thylakoid membranes was performed with white light (1000 µmol photons m^−2^ s^−1^) for 5 min and 15 min, respectively. When TEMPONE EPR spectrum was measured in the presence of α-TOH (1 mM), 0.05% Triton-X 100 was used to prevent precipitation of α-TOH. In C-D, quantification of ^1^O_2_ generated by photosensitization of rose bengal and illumination of thylakoid membranes determined from the height of the central peak of the first derivative of the EPR absorption spectra. Each data point represents the mean ± SD of biological replicates (n = 3).

Singlet oxygen quenching by α-TOH was studied by EPR spectroscopy using TMPD as a spin probe. Oxidation of diamagnetic TMPD by ^1^O_2_ forms paramagnetic TEMPONE detected by EPR spectroscopy. Singlet oxygen was generated either by photosensitization of rose bengal or illumination of thylakoid membranes isolated from Arabidopsis. Addition of TMPD spin probe to rose bengal or thylakoid membranes in the dark did not result in the appearance of TEMPONE EPR spectra (Fig. 2A and B, control trace and Fig. 2C and D, control bar), whereas photosensitization of rose bengal or illumination of thylakoid membranes resulted in the formation of TEMPONE EPR signal (Fig. 2A and B, ^1^O_2_ trace and Fig. 2C and D, ^1^O_2_ bar). When photosensitization of rose bengal or illumination of thylakoid membranes was performed in the presence of α-TOH, TEMPONE EPR signal was significantly suppressed (Fig. 2A and B, ^1^O_2_ + α-TOH trace and Fig. 2C and D, ^1^O_2_ + α-TOH bar). These observations indicate that α-TOH serves as an efficient quencher of ^1^O_2_ generated from the photosensitization of rose bengal or illumination of Arabidopsis thylakoid membranes.

### Formation of α-tocopherol hydroperoxide detected by fluorescence spectroscopy

**Figure 3:**
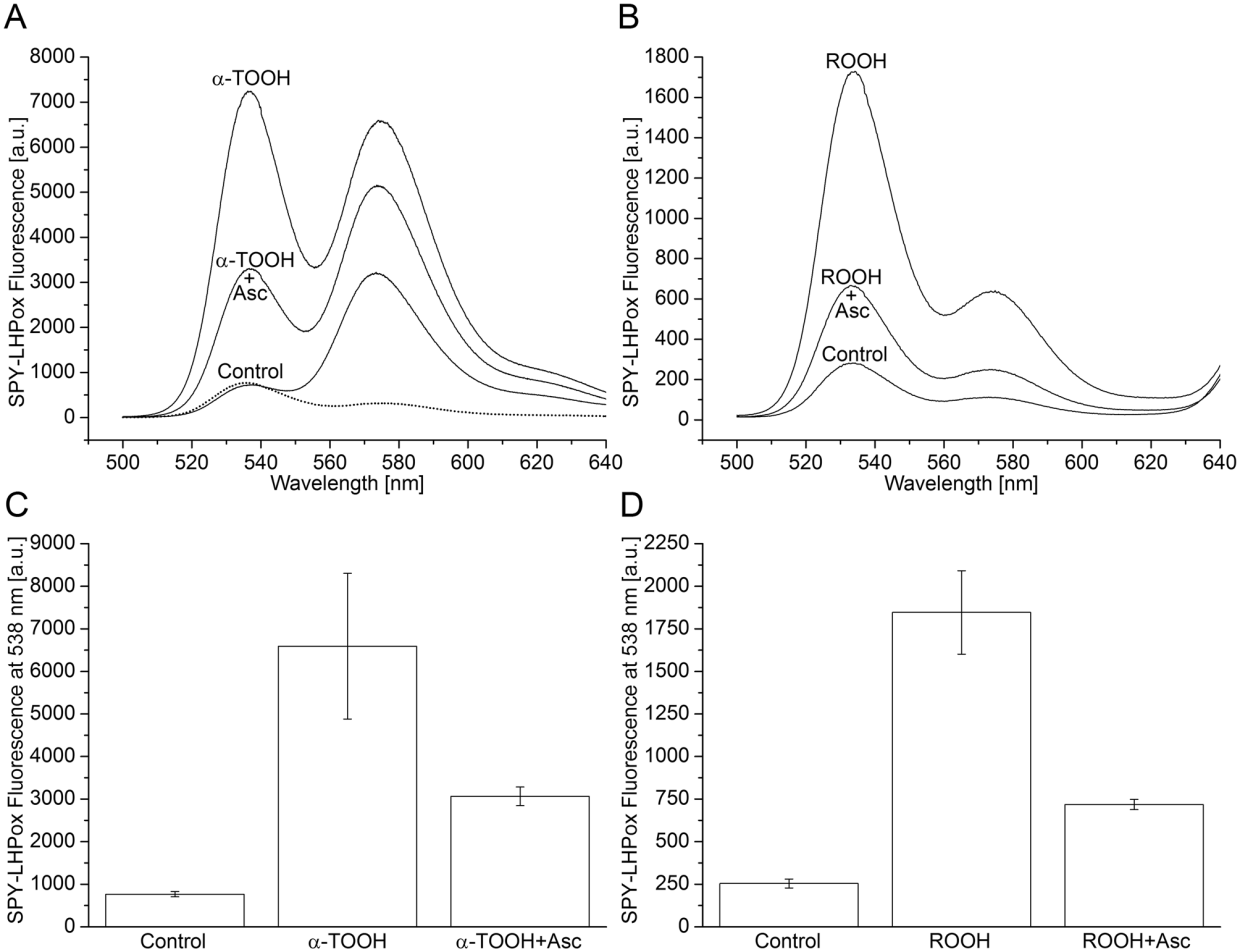
α-Tocopherol hydroperoxide formation in rose bengal (**A, C**) and organic hydroperoxide formation in thylakoid membranes isolated from WT Arabidopsis (**B, D**) monitored by SPY-LHPox fluorescence spectroscopy. In A, SPY-LHPox fluorescence spectra were achieved by the illumination of rose bengal (5 µM) with green light obtained using band-pass interference filter (λ = 560 nm, HBW = 10 nm). In B, SPY-LHPox fluorescence spectra were obtained by the illumination of thylakoid membranes (20 µg Chl ml^−1^) with red light using a long-pass edge interference filter (λ > 600 nm). In C, Quantification of the α-TOOH generated by photosensitization of rose bengal and the effect of sodium ascorbate. In D, Quantification of the ROOH generated by photosensitization of chlorophyll and the effect of α-TOH regeneration by sodium ascorbate. Illumination of rose bengal and thylakoid membranes with SPY-LHP (2.5 µM) was done with green and red light (1000 µmol photons m^−2^ s^−1^) for 5 min and 15 min in the absence or presence of α-TOH (20 µM) and sodium ascorbate (500 µM), respectively. The SPY-LHPox fluorescence intensity at 538 nm was used to quantify the rose bengal-photosensitized formation of α-TOOH and chlorophyll photosensitized formation of ROOH. Each data point represents the mean ± SD of biological replicates (n = 3).

To monitor the formation of α-TOOH after oxidation of α-TOH by ^1^O_2_, fluorescent probe swallow-tailed perylene derivative (SPY-LHP) was used. In this reaction, the oxidation of non-fluorescent SPY-LHP by α-TOOH forms fluorescent oxidized derivative (SPY-LHPox) which provides fluorescence in the green region of the spectrum. Fluorescence spectrum of SPY-LHPox shows fluorescence maximum at 538 nm and 575 nm (Fig. 3A, lower dashed trace) as previously shown by Soh et al.^30^. When SPY-LHPox fluorescence spectrum was measured in the presence of rose bengal and α-TOH in dark, no change was seen in fluorescence at 538 nm whereas, fluorescence at 575 nm was increased due to fluorescence of rose bengal (Fig. 3A, control  trace). Rose bengal-photosensitized ^1^O_2_ formation caused a significant enhancement in SPY-LHPox fluorescence at 538 nm due to α-TOOH formation (Fig. 3A, α-TOOH trace and Fig. 3C, α-TOOH bar). It cannot be excluded that the formation of rose bengal hydroperoxide contributes to the enhancement in SPY-LHPox fluorescence. To confirm that α-TOOH is reduced to α-TOH by ascorbate, the effect of sodium ascorbate on α-TOOH formation was studied. When sodium ascorbate was added to α-TOOH formed by the photosensitized oxidation of α-TOH, the SPY-LHPox fluorescence was decreased (Fig. 3A, α-TOOH + Asc trace and Fig. 3C, α-TOOH + Asc bar). These observations reveal that oxidation of α-TOH by ^1^O_2_ forms α-TOOH which is reduced back to α-TOH by ascorbate. When SPY-LHPox fluorescence spectrum was measured in the thylakoid membranes in dark, SPY-LHPox fluorescence was low due to fluorescence of only SPY-LHP (Fig. 3B, control  trace and Fig. 3D, control bar). When thylakoid membranes were illuminated in the presence of SPY-LHP, increase in the SPY-LHPox fluorescence was observed (Fig. 3B, ROOH  trace and Fig. 3D, ROOH bar). As thylakoid membranes are abundant in lipid and protein, SPY-LHPox fluorescence may be due to the formation of organic hydroperoxides comprising LOOH and POOH. The addition of sodium ascorbate to thylakoid membranes caused a decrease in SPY-LHPox fluorescence (Fig. 3B, ROOH + Asc  trace and Fig. 3D, ROOH + Asc bar) confirming that α-TOOH is reduced to α-TOH by ascorbate and thus it prevents the formation of ROOH. These results indicate that α-TOH prevents oxidation of lipids and proteins by ^1^O_2_ quenching. It is proposed here that detection of α-TOOH from thylakoid membranes might be feasible after separation of α-TOOH from other organic hydroperoxides (LOOH, POOH) and identification by ^1^H-NMR and mass spectrometry^32^.

### Formation of α-tocopherol quinone detected by HPLC

To study the formation of α-TQ by decomposition of α-TOOH, the amount of α-TQ was determined by the reverse-phase HPLC analysis using postcolumn reduction with platinum. Figure 1A and B shows the observed chromatograms with the peak corresponding to α-TQ at retention time 9.1 min. Whereas almost no peak at retention time 9.1 min was detected in rose bengal in dark (Fig. 1A, dark trace), a distinguishable peak was observed in thylakoid membranes (Fig. 1B, dark trace). When rose bengal with α-TOH, and thylakoid membranes were illuminated, peak at retention time 9.1 min was enhanced (Fig. 1A and B, light trace). The concentration of α-TQ in rose bengal (Fig. 1E) and thylakoid membranes (Fig. 1F) in dark was 0.05 ± 0.01 nmol ml^−1^ and 0.39 nmol ml^−1^, where it increases to 3.3 ± 0.04 nmol ml^−1^ and 0.59 ± 0.07 nmol ml^−1^ in light, respectively. These results indicate that α-TOOH decomposes to α-TQ.

### Formation of α-tocopheroxyl radical by EPR spectroscopy

**Figure 4:**
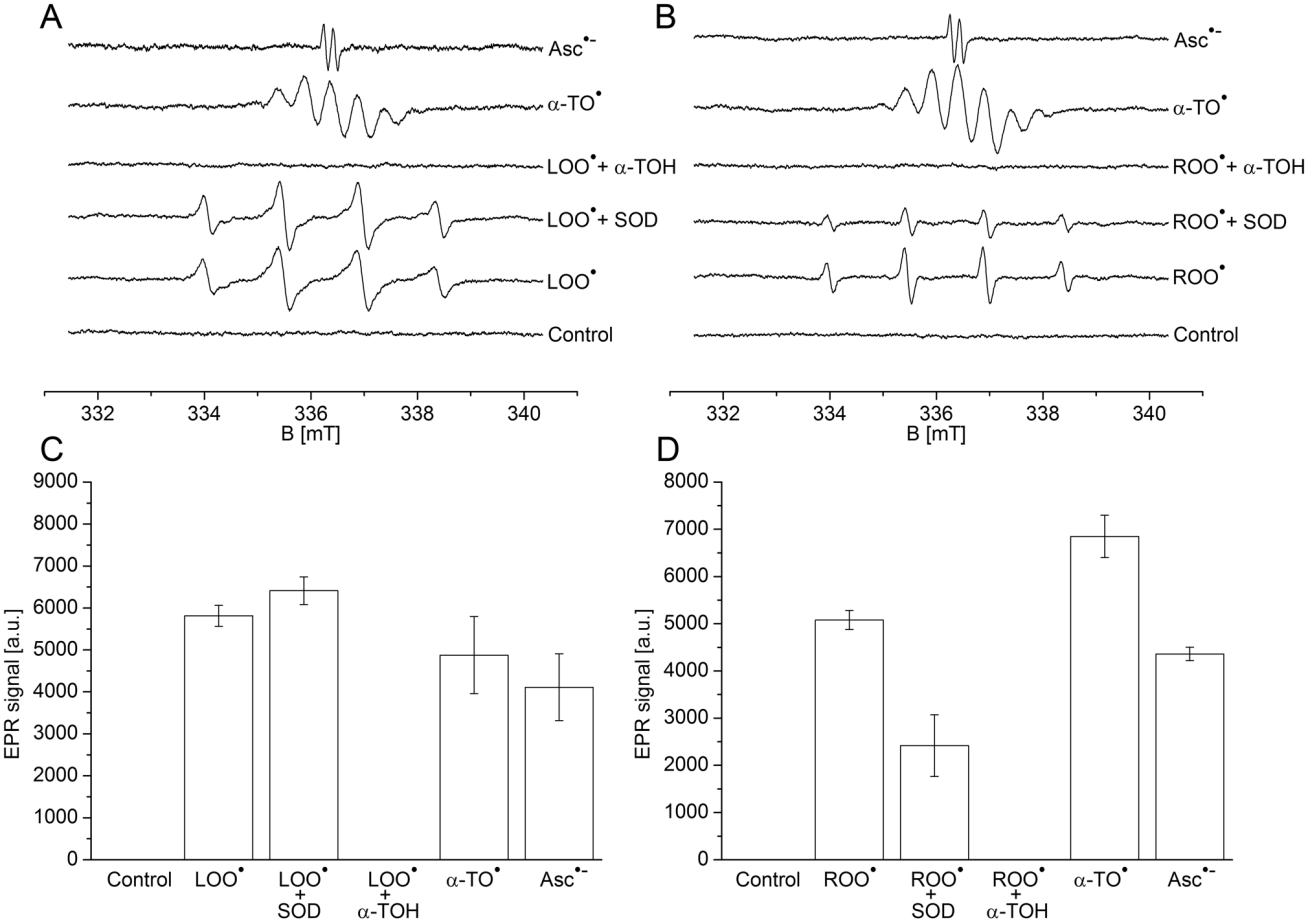
Formation of peroxyl, α-tocopheroxyl and monodehydroascorbate radicals in rose bengal and linolenic acid samples (**A**, **C**) and in thylakoid membranes (**B, D**) monitored by EPR spectroscopy. In A, DMPO-OL adduct EPR spectrum in control (control trace), DMPO-OL adduct EPR spectrum after 5 min of white light illumination (LOO^•^ trace), effect of SOD on DMPO-OL adduct EPR spectrum (LOO^•^ + SOD trace), effect of α-TOH on DMPO-OL adduct EPR spectrum (LOO^•^ + α-TOH trace), α-TO^•^ spectrum (α-TO^•^ trace) and Asc^•−^ spectrum (Asc^•−^ trace) were recorded in rose bengal and linolenic acid samples. In B, DMPO-OR adduct EPR spectrum in control (control trace), DMPO-OR adduct EPR spectrum after 5 min of white light illumination (ROO^•^ trace), effect of SOD on DMPO-OR adduct EPR spectrum (ROO^•^ + SOD trace), effect of α-TOH on DMPO-OR adduct EPR spectrum (ROO^•^ + α-TOH trace), α-TO^•^ spectrum (α-TO^•^ trace) and Asc^•−^ spectrum (Asc^•−^ trace) were recorded in thylakoid membranes. When EPR spectra were measured in the presence of α-TOH, 0.05% Triton-X 100 was used to prevent precipitation of α-TOH. In C, quantification of the LOO^•^ generated by decomposition of LOOH, effect of SOD on LOO^•^  and effect of α-TOH on LOO^•^, α-TO^•^ generated by oxidation of α-TOH during scavenging of LOO^•^ and Asc^•−^ formed during the regeneration of α-TOH from α-TO^•^. In D, quantification of the ROO^•^ generated by decomposition of ROOH effect of SOD on ROO^•^ and effect of α-TOH on ROO^•^, α-TO^•^ generated by oxidation of α-TOH during scavenging of ROO^•^ and Asc^•−^ formed during the regeneration of α-TOH from α-TO^•^. Linolenic acid (250 mM) in the presence of rose bengal (50 µM) or thylakoid membranes (250 µg Chl ml^−1^) were illuminated with white light (1000 µmol photons m^−2^ s^−1^) for 5 min. DMPO-OL/DMPO-OR EPR spectra were measured in the presence of DMPO (50 mM), α-TOH (50 mM), Fe^3+^ (10 mM), SOD (200 U ml^−1^), sodium ascorbate (50 mM) and phosphate buffer (40 mM, pH 7.6). Each data point represents the mean ± SD of biological replicates (n = 3).

To detect the formation of LOO^•^ by decomposition of LOOH in the rose bengal and linolenic acid samples (Fig. 4A) and formation of ROO^•^ by decomposition of ROOH in thylakoid membranes isolated from white light WT Arabidopsis (Fig. 4B), EPR spin-trapping technique was employed using DMPO as the spin-trap compound. Addition of DMPO spin trap to rose bengal and linolenic acid samples or thylakoid membranes in the dark did not result in the appearance of DMPO-OL/DMPO-OR adduct EPR signal (Fig. 4A and B, control trace and Fig. 4C and D, control bar). When rose bengal and linolenic acid samples were illuminated in the presence of Fe^3+^, lipid alkoxyl radical (LO^•^) (DMPO-OL) adduct EPR signal was detected (Fig. 4A, LOO^• ^trace and Fig. 4C, LOO^•^ bar). When thylakoid membranes were illuminated, organic alkoxyl radical (RO^•^) (DMPO-OR) adduct EPR signal was detected (Fig. 4B, ROO^•^ trace and Fig. 4D, ROO^•^ bar). The observation that DMPO-OL adduct EPR signal in the rose bengal and linolenic acid samples was insensitive to SOD excluded contribution of hydroxyl radical (DMPO-OH) adduct (Fig. 4A, LOO^•^ + SOD trace and Fig. 4C, LOO^•^ + SOD bar). In the thylakoid membranes, SOD lowered EPR signal to half (Fig. 4B, ROO^•^ + SOD trace and Fig. 4D, ROO^•^ + SOD bar) revealing that DMPO-OH adduct contributes to overall EPR signal. The addition of α-TOH to the rose bengal and linolenic acid samples or thylakoid membranes caused complete suppression of the formation of LOO^•^/ROO^•^ (Fig. 4A and B, LOO^•^/ROO^•^ + α-TOH trace and Fig. 4C and D, LOO^•^/ROO^•^ + α-TOH bar), while α-TO^•^ was formed (Fig. 4A and B, α-TO^•^ trace and Fig. 4C and D, α-TO^•^ bar). When sodium ascorbate was added together with the α-TOH, α-TO^•^ formed by the oxidation of α-TOH by LOO^•^/ROO^•^ was disappeared and Asc^•−^ appeared (Fig. 4A and B, Asc^•−^ trace and Fig. 4C and D, Asc^•−^ bar). These observations reveal that the oxidation of α-TOH by LOO^•^/ROO^•^ forms α-TO^•^, which is reduced back to α-TOH by ascorbate forming Asc^•−^. These results show that (1) α-TOH scavenges LOO^•^/ROO^•^ in the thylakoid membranes, (2) α-TO^•^ is formed by the oxidation of α-TOH by LOO^•^/ROO^•^ and (3) α-TO^•^ is reduced to α-TOH by sodium ascorbate while ascorbate is oxidized to Asc^•−^.

**Figure 5:**
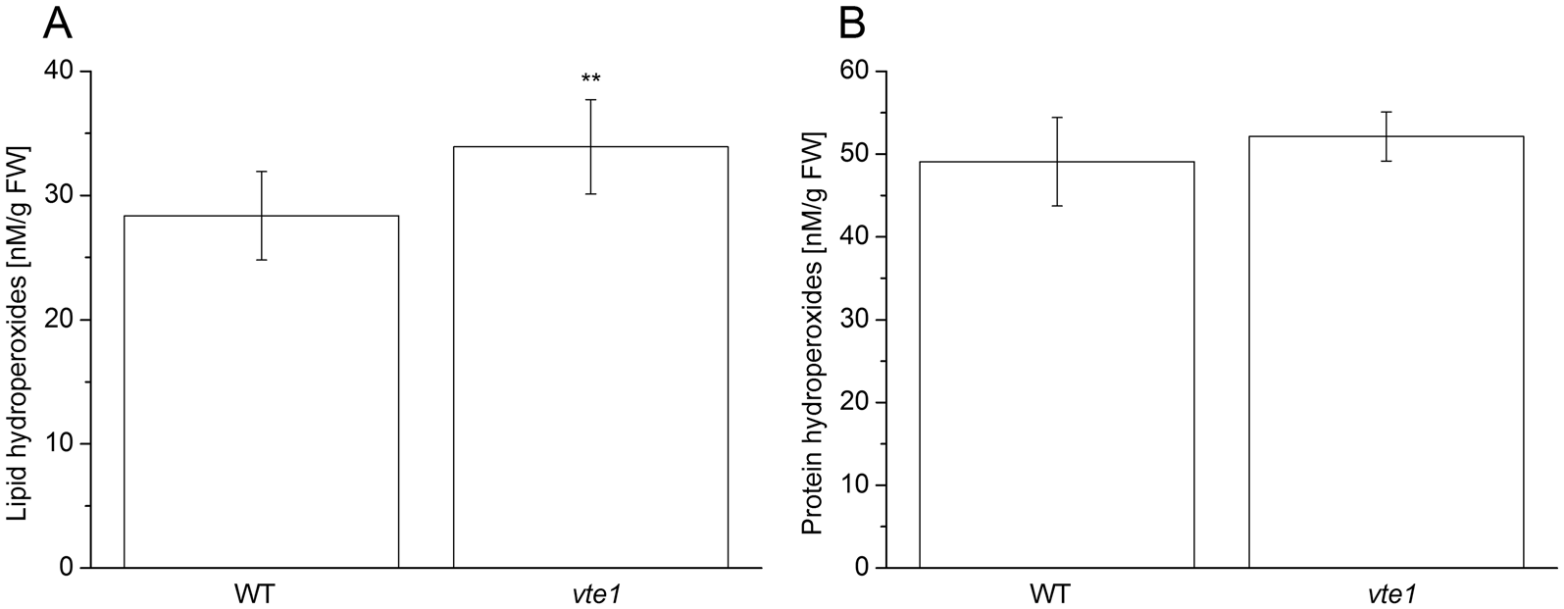
Formation of lipid hydroperoxide (**A**) and protein hydroperoxide (**B**) in WT and *vte1* Arabidopsis quantified by colorimetric ferrous oxidation-xylenol orange (FOX) assay. The concentration of LOOH and POOH was established from the calibration curve obtained using hydrogen peroxide. Each data point represents the mean ± SD of biological replicates (n = 3). A significant difference between high light-exposed WT and *vte1* Arabidopsis plant is indicated by the asterisk ** (Student’s test *p* < 0.001).

### Formation of lipid and protein hydroperoxides by absorption spectroscopy

As SPY-LHP reacts with several types of organic hydroperoxides (LOOH, POOH, α-TOOH), LOOH and POOH were isolated from WT and *vte1* Arabidopsis leaves and quantified using a colorimetric ferrous oxidation-xylenol orange (FOX) assay. In this assay, Fe^3+^ formed by the oxidation of Fe^2+^ by ROOH interacts with xylenol orange (XO) in acidic environments forming an XO–Fe complex with the maximum absorption at 560 nm. The concentration of LOOH (Fig. 5A) and POOH (Fig. 5B) formed in high light illuminated WT and *vte1* Arabidopsis leaves were in the range of several tens of nanomoles. Relatively higher LOOH formation in *vte1* suggests that α-TOH prevents LOOH formation in Arabidopsis leaves at high light.

## Discussion

The antioxidant mechanism of α-TOH was previously examined in many studies^14,33–35^. In the current study, we have shown that consumption of α-TOH caused either by photosensitization of rose bengal (Fig. 1A) or illumination of thylakoid membranes (Fig. 1B) is associated with formation of α-TOH oxidation products: (1) oxidation of α-TOH by ^1^O_2_ forms α-TOOH and (2) oxidation of α-TOH by LOO^•^ forms α-TO^•^. When other antioxidants are present such as ascorbate, oxidation products of α-TOH (α-TOOH and α-TO^•^) are recycled back to α-TOH. Since ascorbate is present in a higher amount than α-TOH, it forms a large reservoir of antioxidant, which could effectively maintain α-TOH restoration. As the most important structural feature of α-TOH in antioxidant activity is chromanol head, oxidation reactions underlying the interaction of ^1^O_2_ and LOO^•^ with electron-rich double bonds in chromanol head are discussed below.

### Oxidation of α-tocopherol by singlet oxygen forms α-tocopherol hydroperoxide

It was formerly shown that the methylene blue-photosensitized oxidation of α-TOH results in the formation of α-TOOH in model systems^36,37^. Detection of α-TOOH using fluorescent probe SPY-LHP showed that ^1^O_2_ photosensitized by rose bengal caused significant formation of α-TOOH (Fig. 3A). It was previously proposed that reaction of α-TOH with ^1^O_2_ occurs via ene reaction^36^. In agreement with this, we propose here that oxidation of α-TOH by ^1^O_2_ occurs by the addition of ^1^O_2_ to double bond carbon on the chromanol head. Chromanol head of α-TOH contains three electron-rich double bonds at the 4th, 6th and 8th positions on the phenol ring which make it a suitable substrate for the ene addition of ^1^O_2_. In the chemical quenching of ^1^O_2_ by α-TOH, the interaction of ^1^O_2_ with electron-rich double bonds at the 8th position of the chromanol head forms α-TOOH by ene reaction.
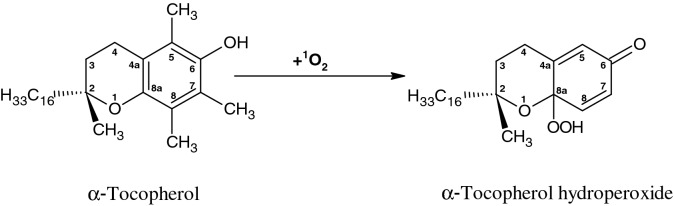


It was formerly shown that α-TOOH formed by the methylene blue-photosensitized oxidation of α-TOH is reduced to α-TOH by ascorbate^37^. Our results of α-TOOH formed by the rose bengal photosensitized oxidation of α-TOH and its reduction to α-TOH by ascorbate (Fig. 3A and C) agrees with the previous reports. Interestingly, the observation that α-TOOH was not fully reduced to α-TOH reveals that α-TOOH decomposes to α-TQ.

### Oxidation of α-tocopherol by lipid peroxyl radical forms α-tocopheroxyl radical

It is well established that major antioxidant function of α-TOH is LOO^•^ scavenging^38^. Scavenging of LOO^•^ by α-TOH is maintained because hydrogen transfer from α-TOH to LOO^•^ is faster than hydrogen transfer from neighboring fatty acid^39,40^. Due to the stereoelectronic features of α-TOH, the stability of α-TO^•^ formed after the hydrogen transfer from α-TOH to LOO^•^ is relatively high. The reaction between α-TOH and LOO^•^ occurs either through concerted hydrogen transfer or via sequential electron transfer followed by proton transfer to form LOOH and α-TO^•^ (Fig. 4A and B, α-TO^•^ trace). The α-TO^•^ can either be reduced back to α-TOH by another cellular reductant such as ascorbate forming Asc^•−^ (Fig. 4A and B, Asc^•−^ trace) or react with another LOO^•^ forming nonradical products. Tocopherol dimers and trimers may be formed during LOO^•^ scavenging as minor products^41^.
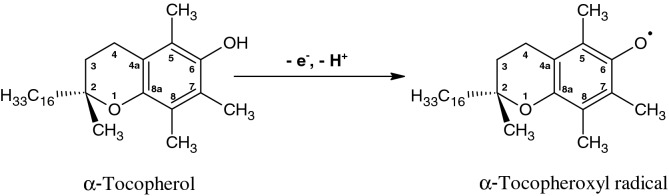


Continuous antioxidant activity provided by α-TOH depends on reductive regeneration of α-TOH from α-TO^•^ by ascorbate^42^. It is well established that α-TO^•^ is reduced by ascorbate to α-TOH while Asc^•−^ is formed^43–47^. Due to continuous regeneration of α-TOH by ascorbate, LOO^•^ scavenging by α-TOH allows suppression of lipid peroxidation at concentrations typically as low as one molecule of α-TOH per thousand phospholipids^34,38,48–52^.

### Relevance for photooxidative stress in Arabidopsis

It was previously demonstrated that herbicide pyrazolynate mediated-inhibition of α-TOH biosynthesis in Chlamydomonas cells under high light stress caused PS II inactivation^53^. Using WT and *vte1* Arabidopsis exposed to high light at low temperature, α-TOH was shown to protect lipids from the photooxidative damage^54,55^. Our observation that formation of LOOH (Fig. 5A) and POOH (Fig. 5B) in WT was lower compared to *vte1* Arabidopsis reveals that α-TOH prevent formation of both LOOH and POOH.

## Conclusion

The aim of this study was to contribute to the understanding of in vitro antioxidant mechanisms of α-TOH against photooxidative stress. Detail description of α-TOOH formation  by ^1^O_2_ chemical quenching and  α-TO^•^ formation by LOO^•^/ROO^•^ scavenging might help to elucidate antioxidant activity of α-TOH in Arabidopsis plants. Mechanism of the cellular antioxidant defense plays a crucial role in regulating the levels of ^1^O_2_ and LOO^•^/ROO^•^ in plants when exposed to a variety of environmental stresses.
